# Supplementary material for: Estimating COVID-19 incidence and prevalence using lateral flow tests in England and Scotland, 2023-2024
Source: Nat Commun. 2026 Jan 16;17:834. doi: 10.1038/s41467-025-67272-9 (PMC12828025; doi:10.1038/s41467-025-67272-9)
Supplement: Supplementary file 1 — Supplementary Information [file 41467_2025_67272_MOESM1_ESM.pdf]

Supplementary Materials

Winter COVID-19 Infection Study Design

Cohort

The cohort sampled for this study were selected from previous participants of the COVID-19 Infection Study (CIS) [1], a household study. Participants were asked if they would consent to being contacted by UKHSA for future studies – those who selected yes were asked to enrol. Individuals eligible for the study lived in private households and were 3 years and older living in England or Scotland. Participants were asked a range of information about themselves and the results of SARS-CoV-2 diagnostic tests. If an individual agreed to participate, they were asked to read the participant information for this study and complete the study consent form. For participants aged under 16 years, a guardian was asked to read, share and discuss the participant information [2] for this study and complete the study consent form.

The study was live from November 2023 to March 2024. Overall, there were 123,243 participants and 426,667 responses. Further information about how this data was collected can be found in the ONS quality and methodology information page for the study [3]. Information for the participants, including how to complete the questionnaire and conduct the tests are found on the ONS website [4]. The participant breakdowns by reference group are given in Supplementary Table (1).

Survey Design

The study had three core surveys, the first “participant” survey collected key identifying information used to link data, such as names, addresses and demographics. The second “main” survey asked further questions relating to individual characteristics (such as job, household size), current and new symptoms, key survey dates (including window start date, LFD taken date, symptom onset date) and lateral flow device (LFD) test results. The third “follow up” survey was given to those who reported a positive test in the “main” survey. The “follow up” survey asked participants to test on alternating days following their first positive test and submit the survey after 15 days.

For each survey participants responded via an online survey portal which guided them through each question. Participants were not offered financial incentives for the study but were given the lateral flow devices as part of the operations.

Participants were grouped into waves, and testing windows. Each participant received 14 tests at the start of the study and responded once to the main survey per wave, a total of 4 times over the course of the study. Each wave ran for approximately 4 weeks, with wave 1 running from 14 November to 14 December 2023, wave 2 from 12 December 2023 to 11 January 2024, wave 3 from 9 January to 8 February 2024 and wave 4 from 6 February to 7 March 2024. To allow for flexible timing in testing and response participants were given a window of 8 days to report their test. Participants were prompted at the start of their testing window and on day 5 to encourage response. Tests taken up to two days before the start of the testing window were allowed to be reported in the main survey.

Data Processing

Data were cleaned and duplicates dropped such that there was one record per participant per wave. Records were removed where linkage information was unavailable, or demographics information not given. This accounted for <1% of the participants and records. Samples were removed where the individual did complete the main survey but did not take an LFD test.

Where a participant did not know their symptom onset date they were prompted to give a placeholder date of the 15th of the most recent month, for both the follow up and main survey. Therefore, values for symptom onset on these dates were assumed as missing. Demographic and participant characteristic information was captured in the first main survey response and not again unless there was a change in circumstance to avoid lengthy surveys. These demographic data were therefore filled forward. Participants who withdrew consent part way through or after the study were excluded from the analysis.

group	strata	n (participants)	% (participants)	n (responses)	% (responses)	% (population)
all		123243		426667		
age	3 to 17	3886	3.15	11083	2.6	20.55
age	18 to 34	4220	3.42	11426	2.68	21.37
age	35 to 44	9959	8.08	29803	6.99	13.49
age	45 to 54	18448	14.97	61061	14.31	12.72
age	55 to 64	30387	24.66	108950	25.54	13.09
age	65 to 74	36813	29.87	133983	31.4	9.77
age	75 and over	19530	15.85	70361	16.49	9
sex	Female	70189	56.95	241721	56.65	50.99
sex	Male	53054	43.05	184946	43.35	49.01
location	North West	13812	11.21	47568	11.15	12
location	East Midlands	8977	7.28	31266	7.33	7.86
location	South East	18782	15.24	65579	15.37	14.94
location	London	18118	14.7	61023	14.3	14.31
location	South West	12732	10.33	44551	10.44	9.19
location	Scotland	11469	9.31	39968	9.37	8.72
location	East of England	13969	11.33	48642	11.4	10.25
location	Yorkshire and The Humber	10634	8.63	36931	8.66	8.84
location	West Midlands	9722	7.89	33590	7.87	9.63
location	North East	5028	4.08	17549	4.11	4.27

Supplementary Table 1: The count (n) and proportion (%) of participants in each strata. The proportions are calculated as the count within a strata divided by the count within a given group.

Additional results and plots

Prevalence

Age group	Minimum prevalence (%)	Maximum prevalence (%)	Minimum prevalence date	Maximum prevalence date
**3 to 17 years**	1.00 (95% CI: 0.608 to 1.60)	4.34 (95% CI: 3.40 to 5.52)	28/02/2024	23/12/2023
**18 to 34 years**	0.725 (95% CI: 0.459 to 1.08)	5.51 (95% CI: 4.51 to 6.62)	02/03/2024	22/12/2023
**35 to 44 years**	0.968 (95% CI: 0.696 to 1.31)	5.98 (95% CI: 5.07 to 6.99)	02/03/2024	22/12/2023
**45 to 54 years**	0.767 (95% CI: 0.572 to 1.01)	4.84 (95% CI: 4.14 to 5.62)	29/02/2024	22/12/2023
**55 to 64 years**	0.501 (95% CI: 0.370 to 0.662)	4.07 (95% CI: 3.48 to 4.72)	02/03/2024	22/12/2023
**65 to 74 years**	0.401 (95% CI: 0.288 to 0.530)	2.82 (95% CI: 2.41 to 3.29)	02/03/2024	22/12/2023
**75 years and over**	0.316 (95% CI: 0.216 to 0.440)	2.52 (95% CI: 2.12 to 2.97)	03/03/2024	22/12/2023

Supplementary Table 2: Age-stratified estimates of the date and value of the maximum and minimum prevalence.

Location	Minimum prevalence (%)	Maximum prevalence (%)	Minimum prevalence date	Maximum prevalence date
**East Midlands**	0.759 (95% CI: 0.546 to 1.09)	4.33 (95% CI: 3.60 to 5.12)	01/03/2024	22/12/2023
**East of England**	0.751 (95% CI: 0.543 to 1.01)	4.85 (95% CI: 4.12 to 5.70)	01/03/2024	22/12/2023
**London**	0.746 (95% CI: 0.506 to 1.01)	5.37 (95% CI: 4.60 to 6.27)	01/03/2024	22/12/2023
**North East**	0.702 (95% CI: 0.492 to 1.07)	3.92 (95% CI: 3.17 to 4.70)	01/03/2024	22/12/2023
**North West**	0.645 (95% CI: 0.462 to 0.894)	4.20 (95% CI: 3.53 to 4.91)	02/03/2024	22/12/2023
**Scotland**	0.584 (95% CI: 0.408 to 0.801)	3.92 (95% CI: 3.28 to 4.60)	01/03/2024	22/12/2023
**South East**	0.768 (95% CI: 0.561 to 1.03)	4.88 (95% CI: 4.18 to 5.71)	01/03/2024	22/12/2023
**South West**	0.789 (95% CI: 0.587 to 1.08)	4.72 (95% CI: 4.01 to 5.59)	29/02/2024	22/12/2023
**West Midlands**	0.723 (95% CI: 0.520 to 1.02)	4.17 (95% CI: 3.51 to 4.96)	01/03/2024	22/12/2023
**Yorkshire and The Humber**	0.659 (95% CI: 0.471 to 0.908)	3.98 (95% CI: 3.31 to 4.72)	01/03/2024	22/12/2023

Supplementary Table 3: Location-stratified estimates of the date and value of the maximum and minimum prevalence.

Supplementary Figure 1: Sex-stratified prevalence

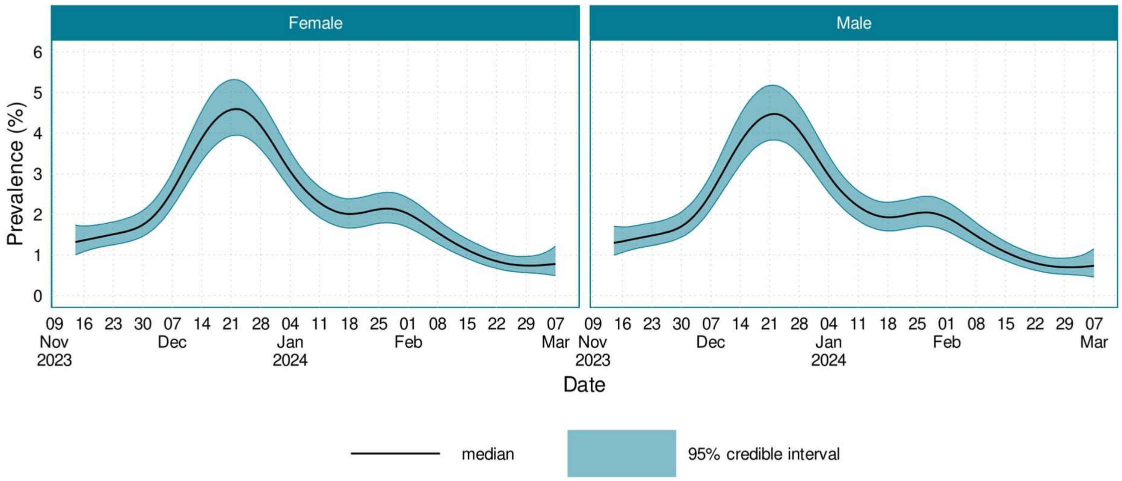

The sex-stratified prevalence of SARS-CoV-2 in England and Scotland between 14th November 2023 and 7th March 2024. Source data are provided as a Source Data file.

Supplementary Figure 2: Reweighted national positivity and prevalence alongside sample positivity

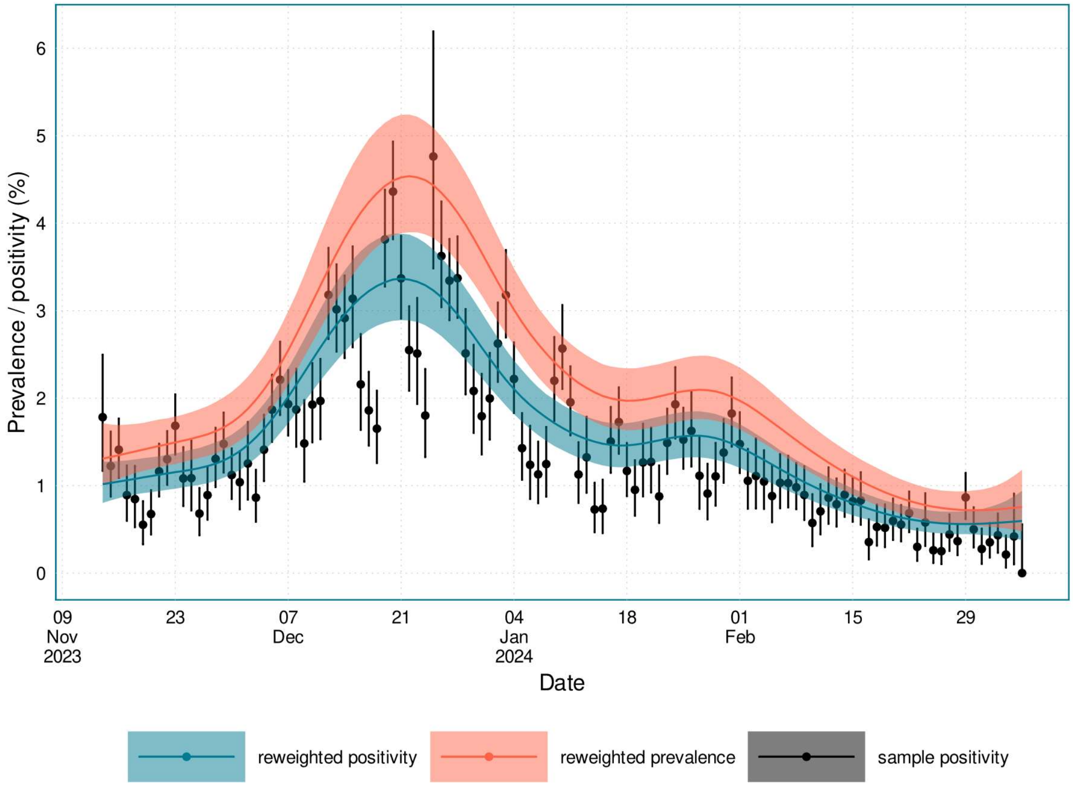

Estimates of the poststratified positivity and prevalence, plotted alongside the daily sample positivity for the combined England and Scotland location. Source data are provided as a Source Data file.

Supplementary Figure 3: Reweighted positivity and prevalence alongside sample positivity, stratified by age group

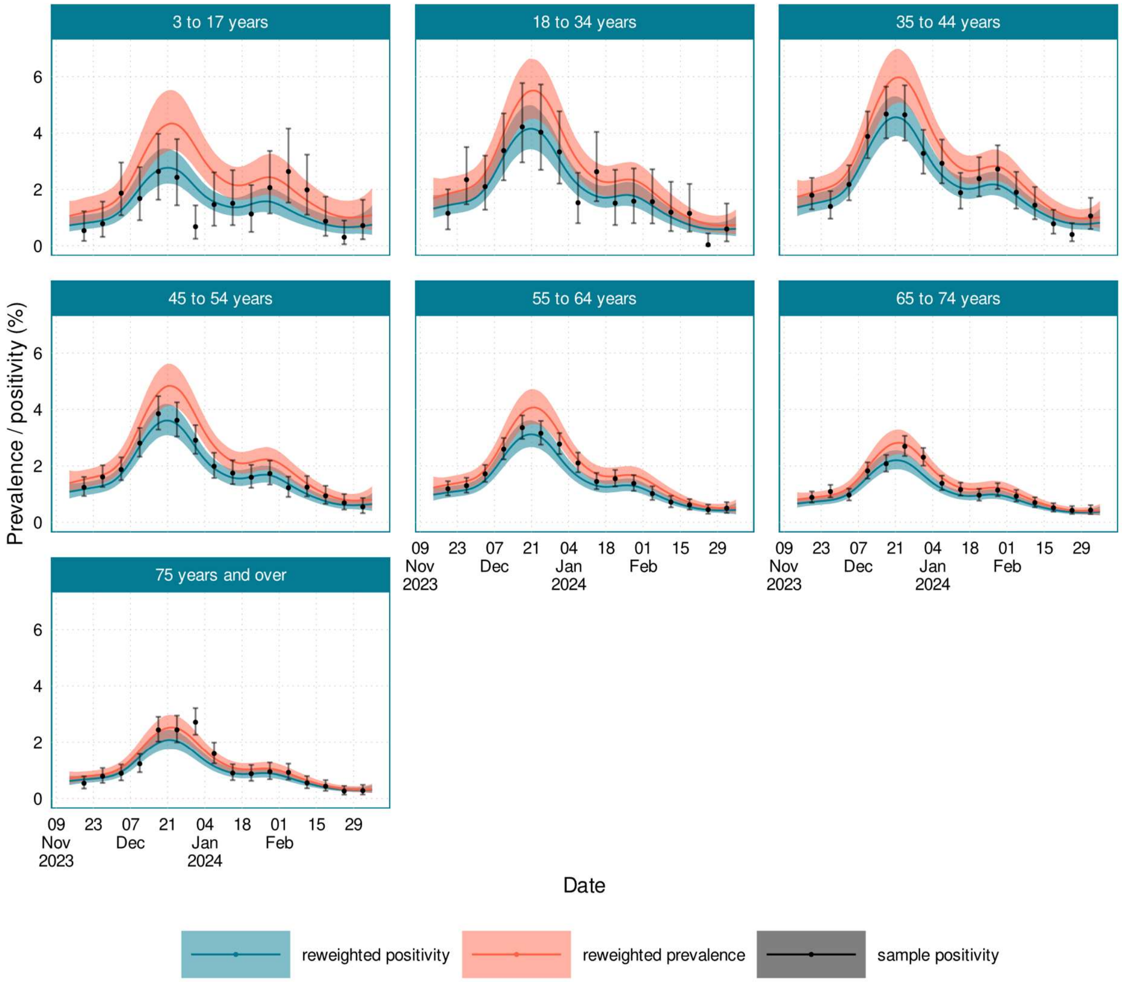

Supplementary Figure 3: Age-stratified estimates of the poststratified positivity and prevalence, plotted alongside the weekly sample positivity. Source data are provided as a Source Data file.

Supplementary Figure 4: Reweighted positivity and prevalence alongside sample positivity, stratified by location

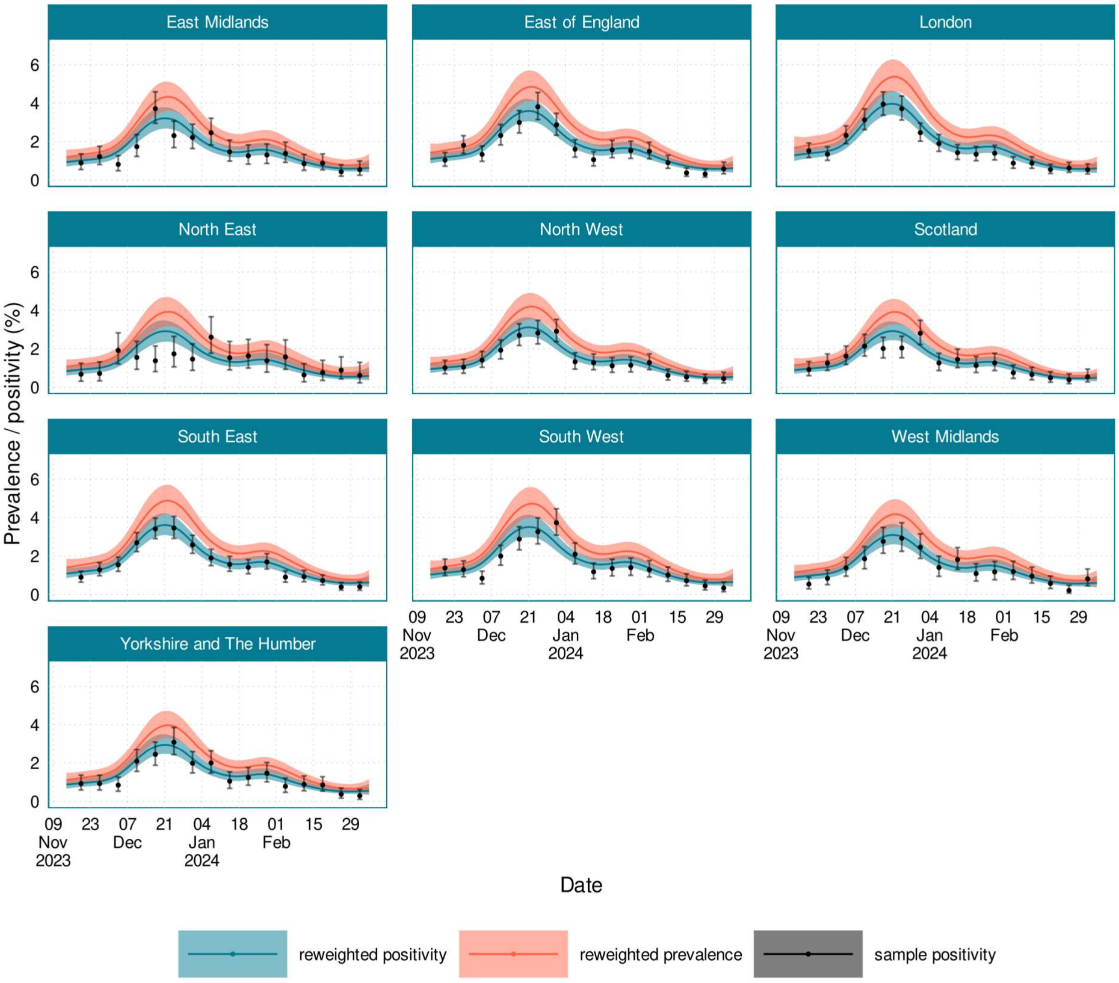

Age-stratified estimates of the poststratified positivity and prevalence, plotted alongside the weekly sample positivity. Source data are provided as a Source Data file.

Supplementary Figure 5: Effect of testing window day

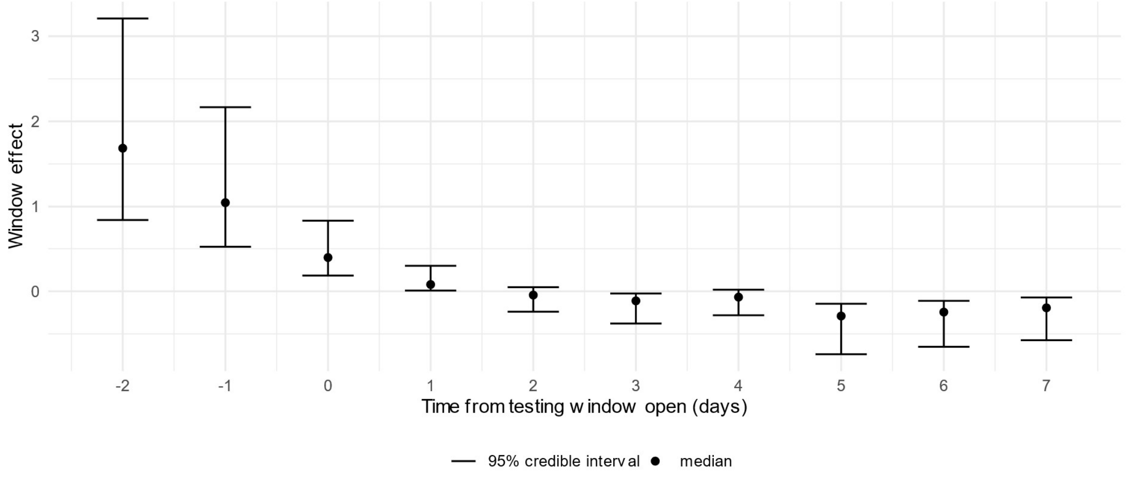

Posterior estimates of the window effect β_i^{window} across different days from window open. Source data are provided as a Source Data file.

Incidence

Age group	Minimum incidence (new infections per 100,000 individuals per day)	Maximum incidence (new infections per 100,000 individuals per day)	Minimum incidence date	Maximum incidence date
**3 to 17 years**	108 (95% CI: 64.2 to 178)	504 (95% CI: 390 to 642)	24/02/2024	18/12/2023
**18 to 34 years**	75.3 (95% CI: 47.6 to 112)	614 (95% CI: 502 to 754)	25/02/2024	17/12/2023
**35 to 44 years**	96.7 (95% CI: 69.3 to 133)	643 (95% CI: 540 to 763)	25/02/2024	17/12/2023
**45 to 54 years**	78.9 (95% CI: 57.2 to 107)	537 (95% CI: 453 to 631)	25/02/2024	17/12/2023
**55 to 64 years**	49.0 (95% CI: 36.1 to 64.9)	432 (95% CI: 364 to 510)	25/02/2024	17/12/2023
**65 to 74 years**	38.1 (95% CI: 27.4 to 51.2)	292 (95% CI: 247 to 347)	25/02/2024	17/12/2023
**75 years and over**	28.4 (95% CI: 19.6 to 39.4)	249 (95% CI: 206 to 299)	25/02/2024	17/12/2023

Supplementary Table 4: Age-stratified estimates of the date and value of peak incidence.

Location	Minimum incidence (new infections per 100,000 individuals per day)	Maximum incidence (new infections per 100,000 individuals per day)	Minimum incidence date	Maximum incidence date
**East Midlands**	77.6 (95% CI: 54.6 to 112)	474 (95% CI: 391 to 573)	24/02/2024	17/12/2023
**East of England**	76.9 (95% CI: 54.3 to 105)	531 (95% CI: 448 to 634)	25/02/2024	17/12/2023
**London**	76.5 (95% CI: 51.6 to 105)	592 (95% CI: 503 to 701)	24/02/2024	17/12/2023

**North East**	71.6<br>(95% CI: 49.7 to 110)	429<br>(95% CI: 344 to 523)	24/02/2024	17/12/2023
**North West**	66.2<br>(95% CI: 46.8 to 90.6)	460<br>(95% CI: 384 to 548)	24/02/2024	17/12/2023
**Scotland**	59.6<br>(95% CI: 41.1 to 82.2)	429<br>(95% CI: 356 to 510)	24/02/2024	17/12/2023
**South East**	78.5<br>(95% CI: 56.8 to 107)	535<br>(95% CI: 452 to 635)	25/02/2024	17/12/2023
**South West**	80.4<br>(95% CI: 58.2 to 111)	517<br>(95% CI: 433 to 618)	25/02/2024	17/12/2023
**West Midlands**	73.9<br>(95% CI: 52.2 to 104)	459<br>(95% CI: 379 to 553)	24/02/2024	17/12/2023
**Yorkshire and The Humber**	67.5<br>(95% CI: 47.4 to 94.4)	437<br>(95% CI: 361 to 526)	25/02/2024	17/12/2023

Supplementary Table 5: Location-stratified estimates of the date and value of peak incidence

Supplementary Figure 6: Sex-stratified incidence

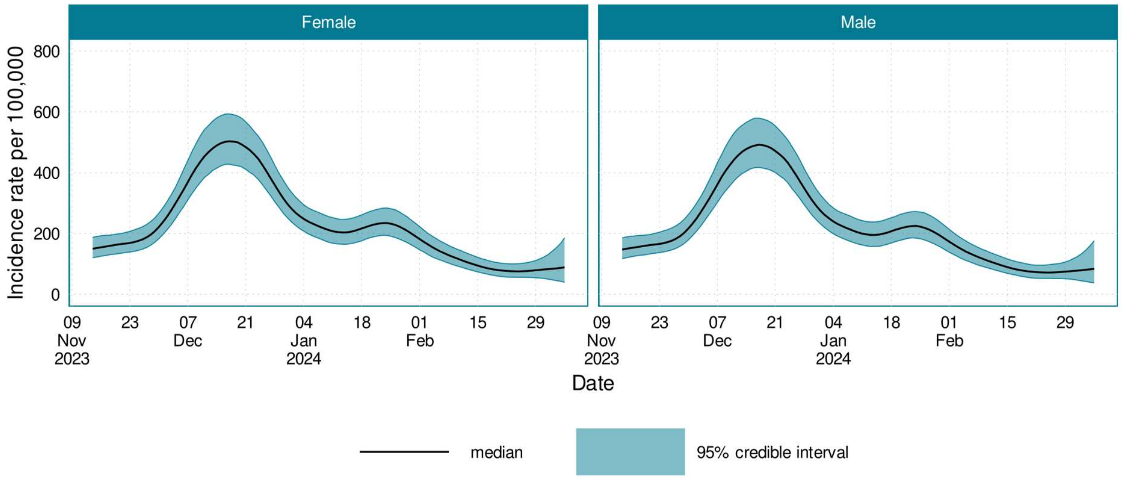

The sex-stratified estimates of the incidence of SARS-CoV-2 infections for 14 November 2023 to 7 March 2024. Source data are provided as a Source Data file.

Average infections per individual

Supplementary Figure 7: The average number of infections per individual over time

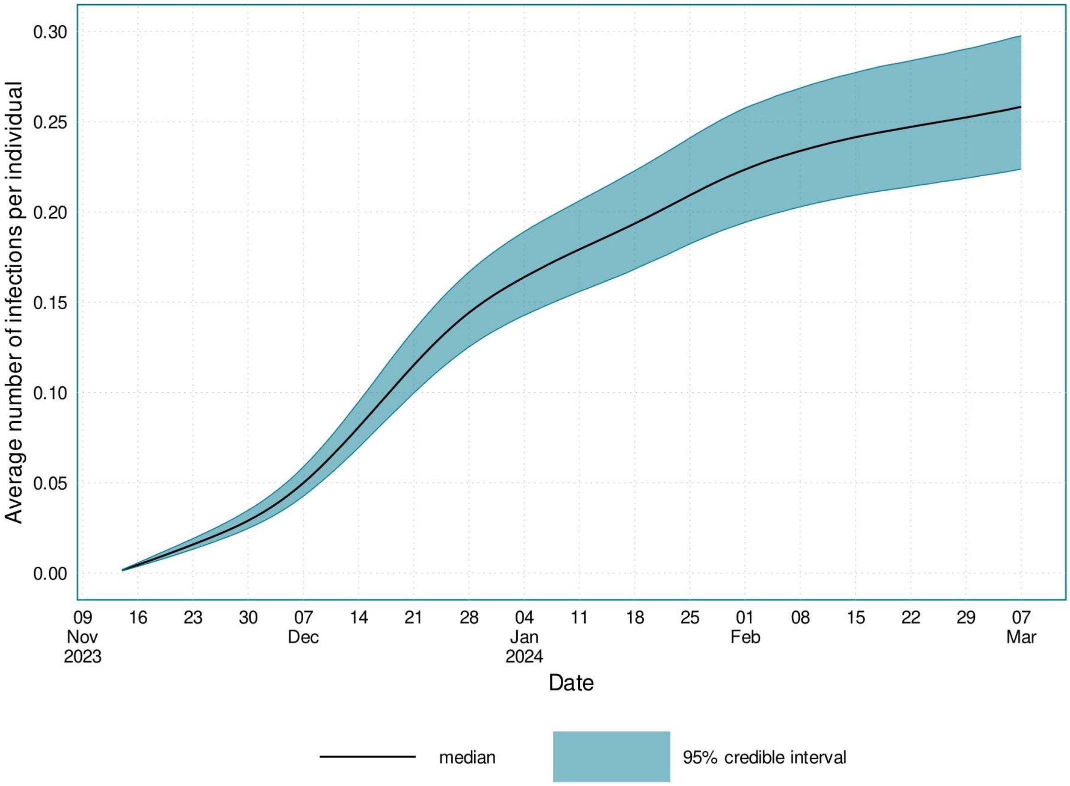

The time-varying average number of infections that had occurred per individual over the study period, 14th November 2023 to 7th March 2024, for England and Scotland. Source data are provided as a Source Data file.

Supplementary Figure 8: The age-stratified average number of infections per individual over time

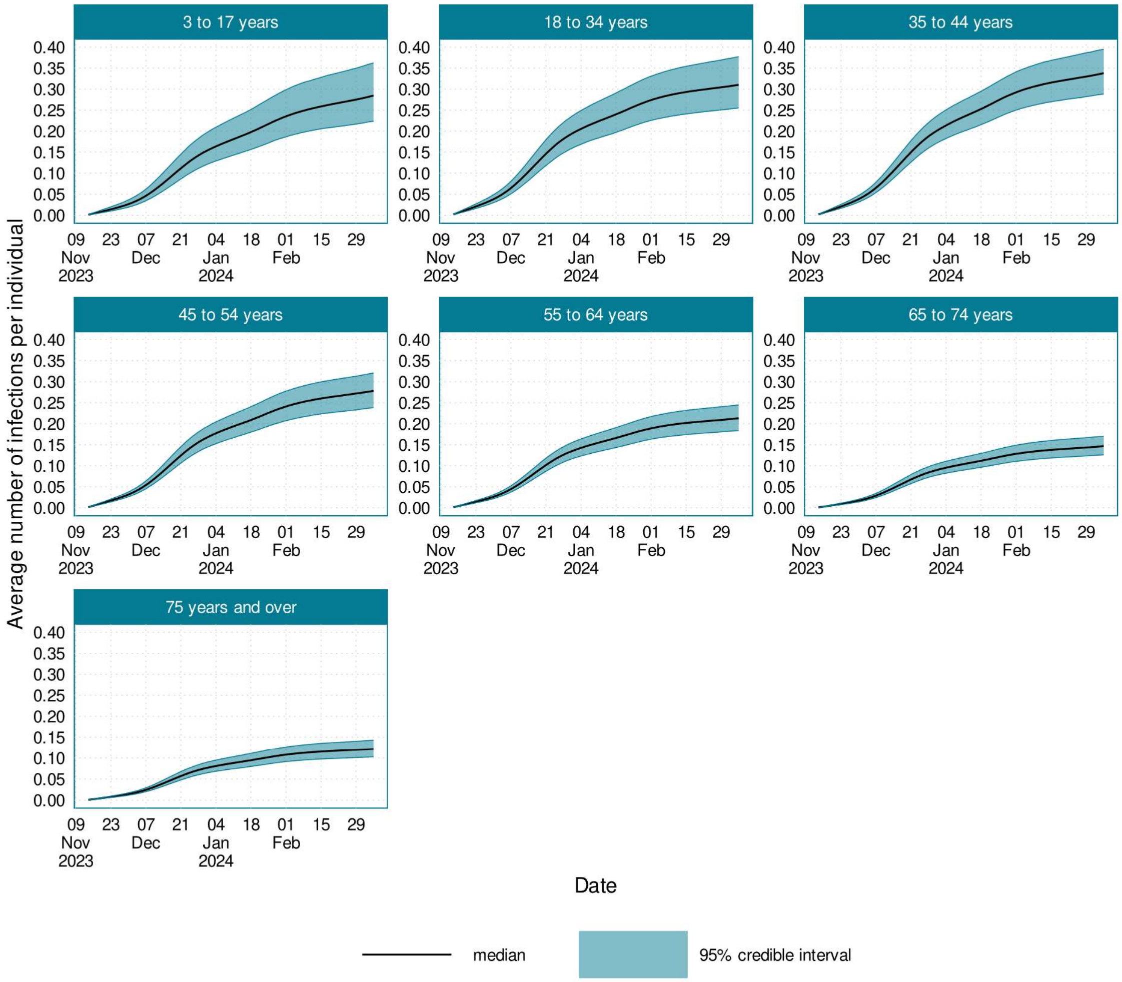

The age-stratified average number of infections that had occurred per individual over the study period, 14th November 2023 to 7th March 2024, for England and Scotland. Source data are provided as a Source Data file.

Supplementary Figure 9: The location-stratified average number of infections per individual over time

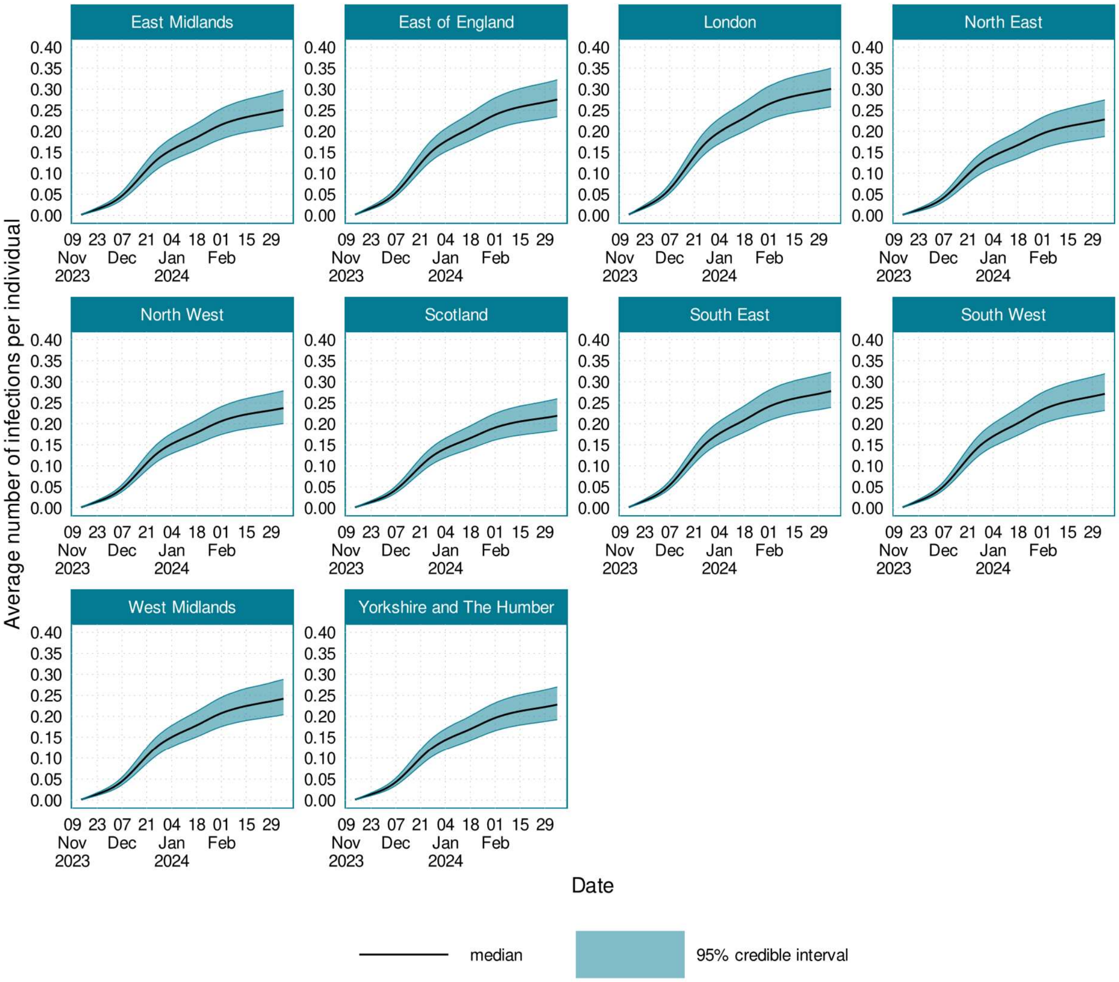

The location-stratified average number of infections that had occurred per individual over the study period, 14th November 2023 to 7th March 2024, for England and Scotland. Source data are provided as a Source Data file.

Supplementary Figure 10: The age-stratified average number of infections per individual over time

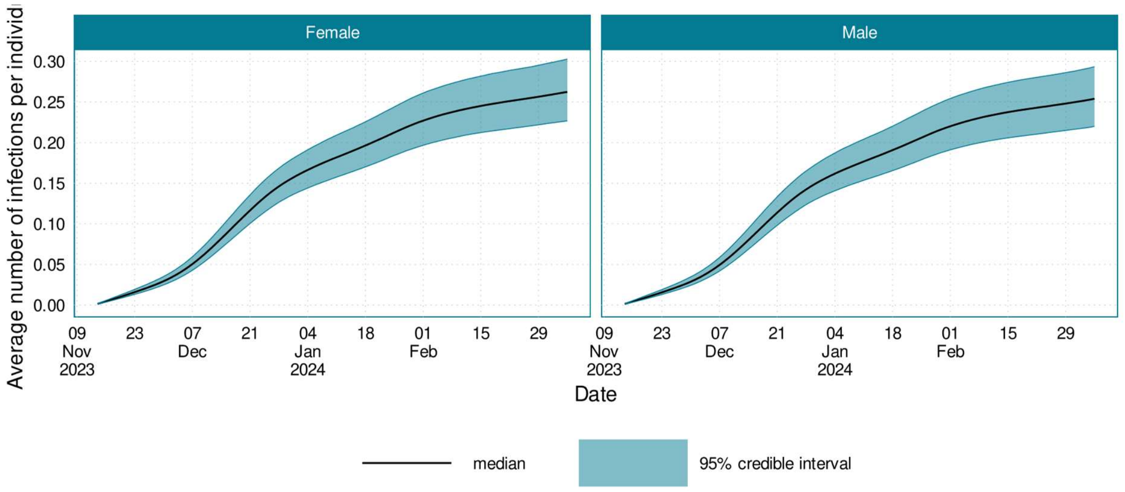

The sex-stratified average number of infections that occurred per individual over the study period, 14th November 2023 to 7th March 2024, for England and Scotland. Source data are provided as a Source Data file.

Sensitivity

Age group	Average sensitivity (%)	Minimum sensitivity (%)	Maximum sensitivity (%)	Maximum sensitivity date	Minimum sensitivity date
**3 to 17 years**	62.0 (95% CI: 55.8 to 67.9)	57.5 (95% CI: 50.8 to 63.9)	68.3 (95% CI: 62.3 to 73.9)	08/12/2023	03/01/2024
**18 to 34 years**	73.5 (95% CI: 68.8 to 77.8)	70.1 (95% CI: 64.8 to 74.9)	78.3 (95% CI: 74.2 to 82.0)	09/12/2023	05/01/2024
**35 to 44 years**	74.3 (95% CI: 71.6 to 76.9)	70.8 (95% CI: 67.6 to 73.9)	79.2 (95% CI: 76.8 to 81.7)	09/12/2023	05/01/2024
**45 to 54 years**	72.3 (95% CI: 70.2 to 74.5)	68.4 (95% CI: 65.7 to 71.0)	77.7 (95% CI: 75.5 to 79.9)	09/12/2023	04/01/2024
**55 to 64 years**	74.1 (95% CI: 72.3 to 75.8)	70.6 (95% CI: 68.4 to 72.7)	79.0 (95% CI: 77.2 to 80.9)	09/12/2023	04/01/2024
**65 to 74 years**	75.3 (95% CI: 73.6 to 77.0)	72.1 (95% CI: 70.1 to 74.0)	79.8 (95% CI: 78.1 to 81.8)	09/12/2023	04/01/2024
**75 years and over**	79.2 (95% CI: 76.9 to 81.3)	76.2 (95% CI: 73.7 to 78.6)	83.4 (95% CI: 81.3 to 85.4)	10/12/2023	05/01/2024

Supplementary Table 6: Posterior summary statistics for the age-stratified population average test sensitivity values over time

Supplementary Figure 11: The location-stratified average LFD test sensitivity over time

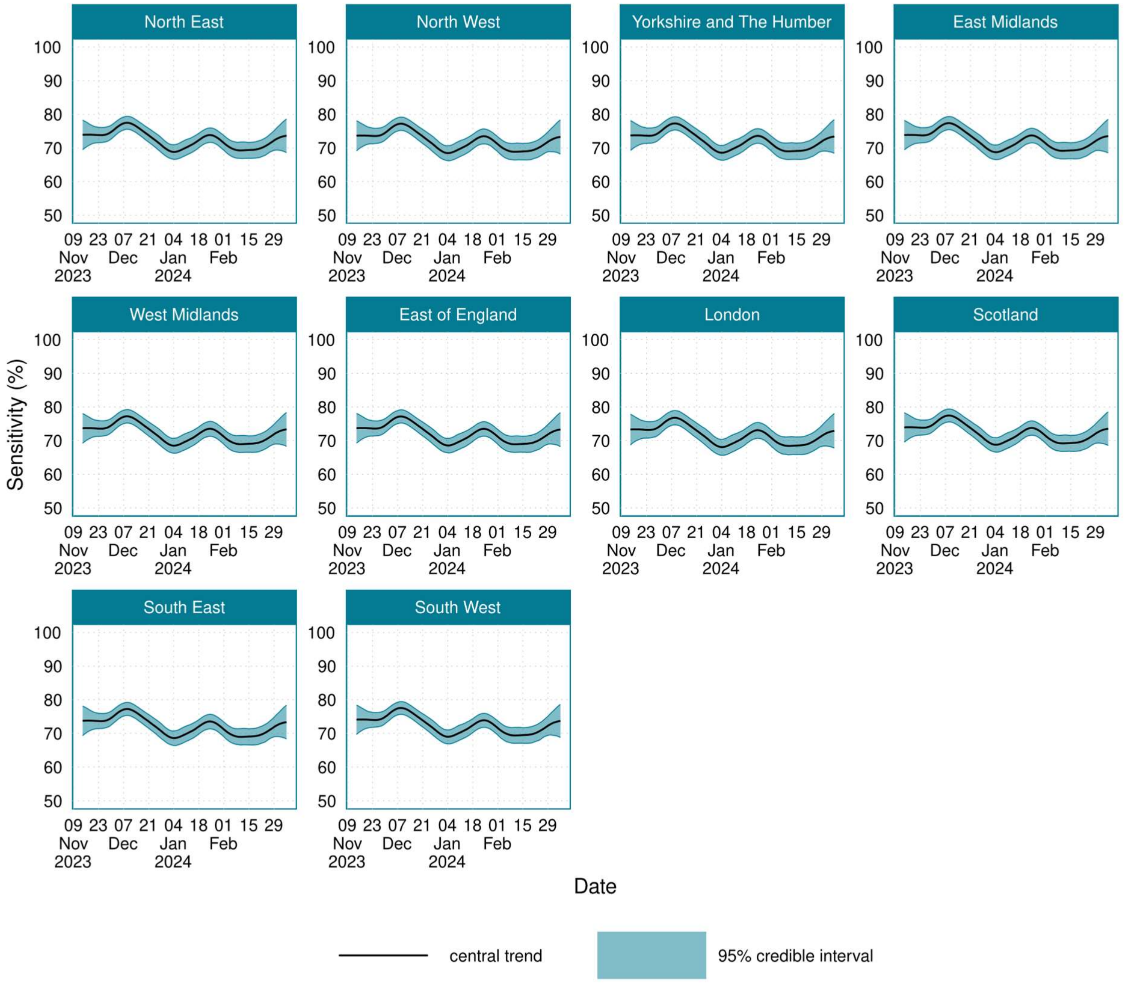

Location-stratified estimates of LFD sensitivity to SARS-CoV-2 infections for 14 November 2023 to 7 March 2024. Source data are provided as a Source Data file.

Location	Average sensitivity (%)	Maximum sensitivity (%)	Minimum sensitivity (%)	Maximum sensitivity date	Minimum sensitivity date
**East Midlands**	72.3 (95% CI: 70.5 to 74.1)	77.4 (95% CI: 75.4 to 79.3)	68.7 (95% CI: 66.5 to 70.8)	09/12/2023	04/01/2024
**East of England**	72.1 (95% CI: 70.3 to 73.9)	77.2 (95% CI: 75.3 to 79.2)	68.6 (95% CI: 66.4 to 70.7)	09/12/2023	04/01/2024
**London**	71.7 (95% CI: 69.6 to 73.7)	76.8 (95% CI: 74.7 to 78.9)	68.1 (95% CI: 65.6 to 70.4)	09/12/2023	04/01/2024
**North East**	72.4 (95% CI: 70.6 to 74.2)	77.5 (95% CI: 75.6 to 79.4)	68.8 (95% CI: 66.6 to 70.9)	09/12/2023	04/01/2024
**North West**	72.1 (95% CI: 70.2 to 73.9)	77.2 (95% CI: 75.2 to 79.1)	68.5 (95% CI: 66.2 to 70.6)	09/12/2023	04/01/2024
**Scotland**	72.4 (95% CI: 70.6 to 74.2)	77.5 (95% CI: 75.6 to 79.4)	68.8 (95% CI: 66.6 to 70.9)	09/12/2023	04/01/2024

**South East**	72.2 (95% CI: 70.4 to 74.0)	77.2 (95% CI: 75.3 to 79.2)	68.6 (95% CI: 66.4 to 70.7)	09/12/2023	04/01/2024
**South West**	72.5 (95% CI: 70.8 to 74.3)	77.5 (95% CI: 75.7 to 79.4)	69.0 (95% CI: 66.9 to 71.0)	09/12/2023	04/01/2024
**West Midlands**	72.1 (95% CI: 70.3 to 74.0)	77.2 (95% CI: 75.2 to 79.2)	68.5 (95% CI: 66.3 to 70.7)	09/12/2023	04/01/2024
**Yorkshire and The Humber**	72.2 (95% CI: 70.4 to 74.0)	77.3 (95% CI: 75.3 to 79.2)	68.6 (95% CI: 66.3 to 70.7)	09/12/2023	04/01/2024

Supplementary Table 7: Posterior summary statistics for the location-stratified population average test sensitivity values over time

Bibliography

[1] Office for National Statistics, "Coronavirus (COVID-19) Infection Survey, UK: 24 March 2023," 24 March 2023. [Online]. Available: https://www.ons.gov.uk/peoplepopulationandcommunity/healthandsocialcare/conditionsanddiseases/bulletins/coronaviruscovid19infectionsurveypilot/24march2023.

[2] Office for National Statistics, "About the Winter Coronavirus (COVID-19) Infection Study," 22 September 2025. [Online]. Available: https://www.ons.gov.uk/surveys/informationforhouseholdsandindividuals/householdandindividualsurveys/wintercoronaviruscovid19infectionstudy/aboutthewintercoronaviruscovid19infectionstudy.

[3] Office for National Statistics, "Winter Coronavirus (COVID-19) Infection Study Quality and Methods Information," 24 June 2024. [Online]. Available: https://www.ons.gov.uk/peoplepopulationandcommunity/healthandsocialcare/conditionsanddiseases/methodologies/wintercoronaviruscovid19infectionstudyqmi. [Accessed 29 July 2024].

[4] Office for National Statistics, "Winter Coronavirus (COVID-19) Infection Study," 26 September 2025. [Online]. Available: https://www.ons.gov.uk/surveys/informationforhouseholdsandindividuals/householdandindividualsurveys/wintercoronaviruscovid19infectionstudy. [Accessed 29 July 2024].
