# Supplementary material for: Estimating COVID-19 incidence and prevalence using lateral flow tests in England and Scotland, 2023-2024
Source: Nat Commun. 2026 Jan 16;17:834. doi: 10.1038/s41467-025-67272-9 (PMC12828025; doi:10.1038/s41467-025-67272-9)
Supplement: Supplementary file 2 — Reporting Summary [file 41467_2025_67272_MOESM2_ESM.pdf]

nature portfolio

Corresponding author(s): Dr Martyn Fyles

Last updated by author(s): 31/7/25

Reporting Summary

Nature Portfolio wishes to improve the reproducibility of the work that we publish. This form provides structure for consistency and transparency in reporting. For further information on Nature Portfolio policies, see our Editorial Policies and the Editorial Policy Checklist.

Statistics

For all statistical analyses, confirm that the following items are present in the figure legend, table legend, main text, or Methods section.

n/a	Confirmed	
☒	☐	The exact sample size (n) for each experimental group/condition, given as a discrete number and unit of measurement
☐	☒	A statement on whether measurements were taken from distinct samples or whether the same sample was measured repeatedly
☒	☐	The statistical test(s) used AND whether they are one- or two-sided<br>*Only common tests should be described solely by name; describe more complex techniques in the Methods section.*
☐	☒	A description of all covariates tested
☐	☒	A description of any assumptions or corrections, such as tests of normality and adjustment for multiple comparisons
☐	☒	A full description of the statistical parameters including central tendency (e.g. means) or other basic estimates (e.g. regression coefficient) AND variation (e.g. standard deviation) or associated estimates of uncertainty (e.g. confidence intervals)
☒	☐	For null hypothesis testing, the test statistic (e.g. F, t, r) with confidence intervals, effect sizes, degrees of freedom and P value noted<br>*Give P values as exact values whenever suitable.*
☐	☒	For Bayesian analysis, information on the choice of priors and Markov chain Monte Carlo settings
☐	☒	For hierarchical and complex designs, identification of the appropriate level for tests and full reporting of outcomes
☒	☐	Estimates of effect sizes (e.g. Cohen's d, Pearson's r), indicating how they were calculated

Our web collection on statistics for biologists contains articles on many of the points above.

Software and code

Policy information about availability of computer code

Data collection	Data collection was performed by the Office for National Statistics.
Data analysis	Data wrangling and results analysis/plotting were performed in R version 4.3.2. A custom Stan model was developed and fitted using Stan version 2.32.2, and cmdstanr version 0.8.1.

For manuscripts utilizing custom algorithms or software that are central to the research but not yet described in published literature, software must be made available to editors and reviewers. We strongly encourage code deposition in a community repository (e.g. GitHub). See the Nature Portfolio guidelines for submitting code & software for further information.

Data

Policy information about availability of data

All manuscripts must include a data availability statement. This statement should provide the following information, where applicable:

- Accession codes, unique identifiers, or web links for publicly available datasets
- A description of any restrictions on data availability
- For clinical datasets or third party data, please ensure that the statement adheres to our policy

UKHSA operates a robust governance process for applying to access protected data that considers:
• the benefits and risks of how the data will be used
• compliance with policy, regulatory and ethical obligations

• data minimisation
• how the confidentiality, integrity, and availability will be maintained
• retention, archival, and disposal requirements
• best practice for protecting data, including the application of 'privacy by design and by default', emerging privacy conserving technologies and contractual controls
Access to protected data is always strictly controlled using legally binding data sharing contracts.
UKHSA welcomes data applications from organisations looking to use protected data for public health purposes.
To request an application pack or discuss a request for UKHSA data you would like to submit, contact DataAccess@ukhsa.gov.uk.

Research involving human participants, their data, or biological material

Policy information about studies with human participants or human data. See also policy information about sex, gender (identity/presentation), and sexual orientation and race, ethnicity and racism.

Reporting on sex and gender	The sex of individuals was self-reported in the main questionnaire. Data on gender was not collected. Overall sample sizes are broken down by sex in the supplementary materials. The results of this study apply to both sexes, as the model includes sex as a covariate and post-stratification is performed to correct for over/under representation of subgroups to produce results that representative of the target population.
Reporting on race, ethnicity, or other socially relevant groupings	The following self-reported categorical variables were used in the analysis: age group in years (3-17, 18-34, 35-44, 45-54, 55-64, 65-74, 75+), location (English ITL 1 statistical regions), and sex (male/female). These variables were chosen as previous studies have demonstrated that these variables affect the key results of the paper: incidence, prevalence, and sensitivity. In addition, estimated population sizes were available for subgroups when broken down by age_group/sex/location, but not when broken down by additional variables or levels. These population sizes of the subgroups are required when adjusting for the over/under representation of certain groups within the sample to produce representative estimates, this requirement limits our ability to explore additional variables or levels.
Population characteristics	Females were slightly over-represented within the data. Younger age groups (aged 3 to 17 years, 18 to 34 years and 35 to 44 years) are under-represented when compared with older age groups (aged 55 to 64 years, 65 to 74 years, and 75 years and over), which are over-represented. Those reporting White ethnicity are over-represented within the sample. Additional details are provided in the Quality and Methodology Information document (https://www.ons.gov.uk/peoplepopulationandcommunity/healthandsocialcare/conditionsanddiseases/methodologies/wintercoronaviruscovid19infectionstudyqmi)
Recruitment	The cohort sampled for this study was selected from previous participants of the COVID-19 Infection Study (CIS), a household study. Participants were asked if they would consent to being contacted by UKHSA for future studies – those who selected yes were asked to enrol in Winter CIS.<br><br>If an individual agreed to participate, they were asked to read the participant information for this study and complete the study consent form. For participants aged under 16 years, a guardian was asked to read, share and discuss the participant information (https://www.ons.gov.uk/surveys/informationforhouseholdsandindividuals/householdandindividualsurveys/wintercoronaviruscovid19infectionstudy/aboutthewintercoronaviruscovid19infectionstudy) for this study and complete the study consent form.<br><br>Individuals eligible for the study lived in private households and were 3 years and older living in England or Scotland. Participants were not offered financial incentives for the study but were given the lateral flow devices as part of the operations.
Ethics oversight	The study received ethical approval from the National Statistician's Data Ethics Advisory Committee.

Note that full information on the approval of the study protocol must also be provided in the manuscript.

Field-specific reporting

Please select the one below that is the best fit for your research. If you are not sure, read the appropriate sections before making your selection.

☒ Life sciences ☐ Behavioural & social sciences ☐ Ecological, evolutionary & environmental sciences

For a reference copy of the document with all sections, see nature.com/documents/nr-reporting-summary-flat.pdf

Life sciences study design

All studies must disclose on these points even when the disclosure is negative.

Sample size	Sample-size calculations were not performed as this was a population study tracking epidemiological metrics. Uncertainty due to sample size was propagated throughout estimates, and the results demonstrate an appropriate level of uncertainty.
Data exclusions	Data were cleaned and duplicates dropped such that there was one record per participant per wave. Records were removed where linkage information was unavailable, or demographics information not given. This accounted for <1% of the participants and records. Samples were removed where the individual did complete the main survey but did not take an LFD test.

Replication	Replication was not required as this was a population study.
Randomization	Randomization was not required as this was a population study.
Blinding	Blinding was not required as this was a population study.

Reporting for specific materials, systems and methods

We require information from authors about some types of materials, experimental systems and methods used in many studies. Here, indicate whether each material, system or method listed is relevant to your study. If you are not sure if a list item applies to your research, read the appropriate section before selecting a response.

Materials & experimental systems

n/a	Involved in the study
☒	☐ Antibodies
☒	☐ Eukaryotic cell lines
☒	☐ Palaeontology and archaeology
☒	☐ Animals and other organisms
☒	☐ Clinical data
☒	☐ Dual use research of concern
☒	☐ Plants

Methods

n/a	Involved in the study
☒	☐ ChIP-seq
☒	☐ Flow cytometry
☒	☐ MRI-based neuroimaging

Plants

Seed stocks	*Report on the source of all seed stocks or other plant material used. If applicable, state the seed stock centre and catalogue number. If plant specimens were collected from the field, describe the collection location, date and sampling procedures.*
Novel plant genotypes	*Describe the methods by which all novel plant genotypes were produced. This includes those generated by transgenic approaches, gene editing, chemical/radiation-based mutagenesis and hybridization. For transgenic lines, describe the transformation method, the number of independent lines analyzed and the generation upon which experiments were performed. For gene-edited lines, describe the editor used, the endogenous sequence targeted for editing, the targeting guide RNA sequence (if applicable) and how the editor was applied.*
Authentication	*Describe any authentication procedures for each seed stock used or novel genotype generated. Describe any experiments used to assess the effect of a mutation and, where applicable, how potential secondary effects (e.g. second site T-DNA insertions, mosiacism, off-target gene editing) were examined.*
